# Supplementary material for: Changing Landscape of Systemic Therapy in Biliary Tract Cancer
Source: Cancers (Basel). 2022 Apr 25;14(9):2137. doi: 10.3390/cancers14092137 (PMC9105885; doi:10.3390/cancers14092137)
Supplement: Supplementary file 1 [file cancers-14-02137-s001.zip › cancers-1677378-supplementary.pdf]

cancers

MDPI

Supplementary Materials: Changing Landscape of Systemic Therapy in Biliary Tract Cancer

Edward Woods, Dat Le, Bharath Kumar Jakka, and Ashish Manne

Table S1. Comparing the trial data of three prominent trials with chemotherapy in the first line (this is not a head–head comparison).

	**GC/NP (Shorff et al. [24]) (High dose vs. low dose)**	**GC (vs Gem) (ABC-02)**	**GC-D (vs GC-placebo) (TOP AZ-1)**
Patient population	BTC	BTC and AC	BTC
PFS (in months)	11.8 (11.4 vs 14.9)	8 (vs 5)	7.2 (vs 5.7)
OS (in months)	19.2 (19.5 vs 15.7)	11.7 (8.1)	12.8 (vs 11.5)
	>G3 AE(%)		
Neutropenia (%)	41 (42 vs 39)	25 (vs 17)	20 (vs 21)
Anemia (%)	16 (19 vs 12)	8 (vs 3)	24 (vs 22)
Thrombocytopenia (%)	13 (16 vs 8)	9 (vs 6)	5 (vs 5)
Fatigue (%)	14	19 (vs 17)	3 (vs 2)
Elevated ALP (%)	4 (3 vs 4)*	NR[#]	Hepatic events -irAE, (0.6 vs 0.3)
Elevated ALT (%)	5 (3 vs 0)**	10	
Peripheral neurophathy (%)	2 (0 vs 4)***	NR	NR
Diarrhea (%)	4 (3 vs 4)****	NR	0.3 vs 0.3

BTC – biliary tract cancers include gall bladder cancers and cholangiocarcinoma; AC- ampullary cancer; GC – gemcitabine and cisplatin; Gem-gemcitabine, NP- nab-paclitaxel, D-durvalumab; NR- not reported; PFS – progression free survival; OS – overall survival; AE- adverse events; irAE – immune related adverse events; *one patient each; ** one patient on the entire group; *** only one patient had neuropathy;**** one patient in each group [#]Abnormal liver function – 17 (vs 27) in GC
